# Gender‐specific prognosis models reveal differences in subarachnoid hemorrhage patients between sexes

**DOI:** 10.1111/cns.14894

**Published:** 2024-08-06

**Authors:** Penglei Xu, Yuchun Liu, Junjie Wang, Anke Zhang, Kaikai Wang, Zefeng Wang, Yuanjian Fang, Xiaoyu Wang, Jianmin Zhang

**Affiliations:** ^1^ Department of Neurosurgery, The Second Affiliated Hospital, School of Medicine Zhejiang University Zhejiang China; ^2^ Clinical Research Center for Neurological Diseases of Zhejiang Province Hangzhou China; ^3^ Department of Neurosurgery, The Fourth Affiliated Hospital, International Institutes of Medicine Zhejiang University School of Medicine Yiwu China; ^4^ Brain Research Institute Zhejiang University Zhejiang China; ^5^ MOE Frontier Science Center for Brain Science & Brain‐Machine Integration Zhejiang University Zhejiang China

**Keywords:** female, nomograms, prognosis, sex characteristics, subarachnoid hemorrhage

## Abstract

**Background:**

Subarachnoid hemorrhage (SAH) represents a severe stroke subtype. Our study aims to develop gender‐specific prognostic prediction models derived from distinct prognostic factors observed among different‐gender patients.

**Methods:**

Inclusion comprised SAH‐diagnosed patients from January 2014 to March 2016 in our institution. Collected data encompassed patients' demographics, admission severity, treatments, imaging findings, and complications. Three‐month post‐discharge prognoses were obtained via follow‐ups. Analyses assessed gender‐based differences in patient information. Key factors underwent subgroup analysis, followed by univariate and multivariate analyses to identify gender‐specific prognostic factors and establish/validate gender‐specific prognostic models.

**Results:**

A total of 929 patients, with a median age of 57 (16) years, were analyzed; 372 (40%) were male, and 557 (60%) were female. Differences in age, smoking history, hypertension, aneurysm presence, and treatment interventions existed between genders (*p* < 0.01), yet no disparity in prognosis was noted. Subgroup analysis explored hypertension history, aneurysm presence, and treatment impact, revealing gender‐specific variations in these factors' influence on the disease. Screening identified independent prognostic factors: age, SEBES score, admission GCS score, and complications for males; and age, admission GCS score, intraventricular hemorrhage, treatment interventions, symptomatic vasospasm, hydrocephalus, delayed cerebral ischemia, and seizures for females. Evaluation and validation of gender‐specific models yielded an AUC of 0.916 (95% CI: 0.878–0.954) for males and 0.914 (95% CI: 0.885–0.944) for females in the ROC curve. Gender‐specific prognostic models didn't significantly differ from the overall population‐based model (model 3) but exhibited robust discriminative ability and clinical utility.

**Conclusion:**

Variations in baseline and treatment‐related factors among genders contribute partly to gender‐based prognosis differences. Independent prognostic factors vary by gender. Gender‐specific prognostic models exhibit favorable prognostic performance.

## Graphical abstract

Gender‐specific prognostic models were developed based on distinct factors among SAH patients. Variations in baseline characteristics and treatments partially contribute to gender‐based prognosis differences. Independent prognostic factors vary by gender. Gender‐specific prognostic models demonstrated robust discriminative ability, highlighting the importance of considering gender‐specific factors in SAH prognosis.

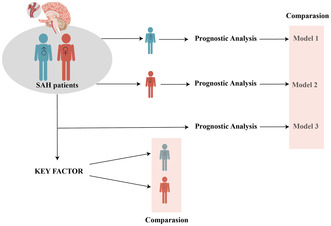

## 1 INTRODUCTION

Spontaneous subarachnoid hemorrhage (SAH) presents a high fatality and disability rate, affecting younger individuals compared to common ischemic strokes.^1^ And according to previous reports, nearly 500,000 patients suffered SAH annually worldwide.^2^ While advancements in clinical interventions and neurocritical care have reduced SAH mortality from 50% to 35%, only 30% of patients fully regain independence.^3^, ^4^, ^5^ Aneurysm rupture, accountable for 70%–85% of SAH cases, notably predisposes females to aneurysmal SAH.^6^, ^7^, ^8^, ^9^, ^10^

Gender emerges as an independent predictor for post‐SAH complications such as hydrocephalus and delayed cerebral ischemia (DCI), potentially influencing prognosis.^11^, ^12^, ^13^ Recent analyses confirmed prognosis disparities among genders in SAH patients.^14^, ^15^ However, the specific factors driving these gender‐related prognosis differences remain unclear. Differences in prognosis stem from two aspects: (1) physiological and biochemical variations due to gender, including hormonal distinctions,^16^ and (2) disparities in baseline data, clinical diagnosis, and treatment among patient populations,^17^ although these distinctions may not have strict boundaries.

Our study aims to explore these gender‐based differences among SAH patients, utilizing subgroup analyses to investigate specific factors' roles. Employing univariable and multivariable analysis, we intend to identify independent prognostic factors per gender, confirming gender‐based variations. Finally, we aim to construct and validate gender‐specific independent prognostic models based on identified gender‐specific prognostic factors, visualizing them as a nomogram. We anticipate these findings will enhance our understanding of gender‐related prognosis differences, potentially offering insights to enhance clinical interventions.

## 2 METHODS

### 2.1 Study population and follow‐up

We conducted a retrospective review of consecutive patients admitted to our institution and diagnosed with SAH between January 2014 and March 2016. SAH diagnosis was confirmed through computer tomography (CT) and lumbar puncture results post‐administration. Demographic, clinical, and imaging data were examined. Inclusion criteria: (1) patients who underwent CT or digital subtraction angiography (DSA) within 3 days after the onset of symptoms; (2) patients with a follow‐up record for 3 months post‐discharge. Exclusion criteria: (1) patients previously diagnosed with traumatic brain injury; (2) patients with unavailable radiological imaging data; (3) patients whose diagnosis of SAH was subsequently corrected; (4) patients had a previous subarachnoid hemorrhage. Subsequently, these patients were included in our analysis.

### 2.2 Patient management and grading

Patients admitted to our institution received management in accordance with the SAH guidelines from the Neurocritical Care Society and the American Heart Association. Nimodipine was regularly administered to prevent vasospasm while maintaining euvolemia. Baseline characteristics collected included: age, sex, number of children, smoking condition, anticoagulant use, hypertension, diabetes, and hyperlipidemia. Additionally, menopausal status and number of children were collected for female patients. The severity of SAH was assessed using the World Federation of Neurological Societies Scale (WFNS) grade, Glasgow coma scale (GCS), and Hunt‐Hess (HH) grade. Radiological information includes: the modified Fisher scale (mFS), subarachnoid hemorrhage early brain edema score (SEBES), intraventricular hemorrhage (IVH), intracerebral hematoma, and midline shift. DSA was reviewed to determine the cause of SAH. Coiling or clipping was performed to secure the detected intracranial aneurysm if feasible. Craniotomy for hematoma evacuation or observation was applied to patients unsuitable for coiling or clipping, categorized as “other intervention” in our analysis. SAH‐related complications were recorded, including: rebleeding, hydrocephalus, symptomatic vasospasm, DCI, and epilepsy. Clinical outcomes were assessed using the Glasgow outcome score (GOS) at 3 months post‐discharge via telephone interviews or outpatient follow‐ups. Prognosis was categorized as favorable (GOS = 4, 5) or poor (GOS = 1–3) outcomes.

### 2.3 Grouping of patients

Patients were stratified by gender into two groups for comparative analysis of general clinical data. Subsequently, for each gender, univariate analysis was separately conducted to explore potential gender‐specific prognostic predictors. Prognosis models specific to each gender were developed and validated within their respective gender groups for performance assessment. To ascertain if these gender‐specific models outperform the prognosis model built on the entire cohort, we identified independent prognostic factors of the entire patient cohort and developed a prognosis model for all SAH patients. With an adequate sample size in the entire cohort, we divided the patients randomly in a 7:3 ratio, using 70% of patients for factor selection and model development, and the entire cohort for validation to evaluate model performance. Stratified random sampling was utilized to ensure no significant discrepancies in features between groups.

### 2.4 Nomogram construction and validation

Variables with *p* < 0.05 in univariate analysis were included in the subsequent multivariate analysis using forward stepwise logistic regression analysis. Variables with *p* < 0.05 in the multivariate analysis were chosen as independent gender‐specific prognostic predictors for each gender in the prognosis model. Prognosis models were visualized as nomograms. Discrimination was evaluated using the area under the curve (AUC) of receiver operating characteristic curve (ROC). Consistency between predicted probabilities and observed probabilities was evaluated through calibration curves. The clinical utility of nomograms was evaluated using decision curve analysis (DCA).

### 2.5 Statistical methods

Statistical analyses were conducted using R software (Version 4.1.2, The R Foundation for Statistical Computing) and IBM‐SPSS (Version 26.0, SPSS Inc., Armonk, NY). Continuous variables were assessed for normality using the Kolmogorov–Smirnov (KS) test. Descriptive statistics were presented as mean ± SD for variables with a normal distribution, and non‐paired Student's *t*‐tests were utilized for statistical analysis. For variables not conforming to a normal distribution, median (IQR) was reported, and non‐parametric tests were applied. Categorical variables underwent analysis using either *χ* ^2^ or Fisher's exact tests. Multivariable analysis was conducted using forward stepwise logistic regression.

## 3 RESULTS

### 3.1 Demographics and clinical characteristics of the study population

Initially 1180 patients diagnosed with SAH were assessed, following which 251 individuals were excluded (Figure S1). Eventually, 929 patients, with a median age of 57 (16) years, were included for subsequent analysis, comprising 557 females (60.0%) and 372 males (40.0%). Patient baseline data are depicted in Table 1. There were no significant differences observed in functional scores at 3 months between patients of different genders. However, notable discrepancies emerge in age, smoking status, hypertension situation, aneurysm detection rates, and treatment methods. This prompted our interest in exploring how these distinctions influence genders differently. Consequently, among the many differing factors, we chose hypertension, aneurysm, and treatment method—considered highly impactful on SAH disease—for subgroup analysis, aiming to clarify their effects (Tables S1–S3). Results showed that among female SAH patients with a history of hypertension, there was an increased susceptibility to midline shift on the initial CT post‐onset (*p* = 0.014), a higher probability of postoperative hydrocephalus (*p* = 0.036), and a greater tendency toward unfavorable prognosis (*p* = 0.014). Male patients with aneurysmal SAH displayed a higher rate of initial CT‐observed IVH compared to non‐aneurysmal SAH patients (*p* < 0.001). Females undergoing craniotomy for aneurysm clipping had a higher likelihood of poor outcomes than those choosing coiling (*p* < 0.001), a disparity not seen among male patients. Moreover, gender‐specific differences in aneurysm characteristics among patients with detected aneurysms are delineated in Table 2.

**TABLE 1:** Baseline comparison between genders (preoperative).

Variable	Male	Female	*p*
	*N* (%)	*N* (%)	
No.	372	557	
Age (y.o)	53 (15)	58 (16)	**<0.001** ^a^
Number of children	2 (1)	2 (2)	**<0.001** ^a^
Smoking period (y)	20 (30)	0 (0)	**<0.001** ^a^
Age			
<55	195 (52.4%)	224 (40.2%)	**<0.001**
≥55, <75	168 (45.2%)	296 (53.1%)	
≥75	9 (2.4%)	37 (6.6%)	
Smoking situation			
Current	252 (67.7%)	17 (3.1%)	**<0.001**
Stopped	14 (3.8%)	3 (0.5%)	
Never	106 (28.5%)	537 (96.4%)	
Hypertension	104 (28%)	225 (40.4%)	**<0.001**
Hyperlipidemia	108 (29%)	195 (35%)	0.067
Diabetes	10 (2.7%)	30 (5.4%)	0.069
Antiplatelet drugs	1 (0.3%)	3 (0.5%)	0.653
Heart disease	4 (1.1%)	7 (1.3%)	1.000
Intracranial hematoma	45 (12.1%)	77 (13.8%)	0.506
IVH	135 (36.3%)	192 (34.5%)	0.618
Midline shift	14 (3.8%)	28 (5%)	0.455
Aneurysm	271 (72.8%)	455 (81.7%)	**0.002**
WFNS			
I	273 (73.4%)	390 (70%)	0.484
II	26 (7%)	45 (8.1%)	
III	7 (1.9%)	8 (1.4%)	
IV	43 (11.6%)	63 (11.3%)	
V	23 (6.2%)	51 (9.2%)	
HH			
1	63 (16.9%)	71 (12.7%)	0.185
2	209 (56.2%)	307 (55.1%)	
3	46 (12.4%)	88 (15.8%)	
4	44 (11.8%)	67 (12%)	
5	10 (2.7%)	24 (4.3%)	
mFS			
0	14 (3.8%)	35 (6.3%)	0.085
1	71 (19.1%)	91 (16.3%)	
2	71 (19.1%)	138 (24.8%)	
3	79 (21.2%)	103 (18.5%)	
4	137 (36.8%)	190 (34.1%)	
SEBES			
0	119 (32%)	162 (29.1%)	0.154
1	39 (10.5%)	64 (11.5%)	
2	58 (15.6%)	83 (14.9%)	
3	46 (12.4%)	48 (8.6%)	
4	110 (29.6%)	200 (35.9%)	
Admission GCS			
0 (15)	276 (74.2%)	390 (70%)	0.437
1 (13–14)	29 (7.8%)	48 (8.6%)	
2 (9–12)	28 (7.5%)	42 (7.5%)	
3 (3–8)	39 (10.5%)	77 (13.8%)	
Intervention			
Clipping	150 (40.3%)	218 (39.1%)	**0.001**
Coiling	113 (30.4%)	226 (40.6%)	
Others	109 (29.3%)	113 (20.3%)	
Drainage			
LCFD	109 (29%)	143 (25.7%)	0.236
EVD	10 (2.4%)	14 (2.5%)	
Complications	142 (38.2%)	193 (34.6%)	0.305
Hydrocephalus	55 (14.8%)	66 (11.8%)	0.229
Vasospasm	102 (27.4%)	126 (22.6%)	0.112
DCI	92 (24.7%)	127 (22.8%)	0.548
Epilepsy	7 (1.9%)	8 (1.4%)	0.793
Rebleeding	4 (1.1%)	12 (2.2%)	0.326
GOS at 3 months			
Favorable (4–5)	293 (78.8%)	443 (79.5%)	0.805
Poor (1–3)	79 (21.2%)	114 (20.5%)	

*Note*: Age, number of children, and years of smoking are continuous variables, expressed as median (IQR). Bolded numbers indicate *p*‐values <0.05, signifying statistical significance.

^a^ These variables exhibit a non‐normal distribution; hence, non‐parametric tests are chosen for statistical analysis. Due to rounding, some percentage data may not sum up to 100%.

**TABLE 2:** Aneurysm characteristics comparison between genders.

Variable	Female	Male	*p*
	*N* (%)	*N* (%)	
Size (maximum diameter)			
<5 mm	239 (62.1%)	150 (62.5%)	0.674
≥5 mm, <7 mm	100 (26%)	57 (23.8%)	
≥7 mm, <10 mm	32 (8.3%)	26 (10.8%)	
≥10 mm	14 (3.6%)	7 (2.9%)	
Multiple aneurysms			
No	414 (91.6%)	259 (96.3%)	**0.022**
Yes	38 (8.4%)	10 (3.7%)	
Position			
Anterior circulation	234 (51.8%)	206 (76.6%)	**<0.001**
Posterior circulation	180 (39.8%)	53 (19.7%)	

*Note*: Bolded numbers indicate *p*‐values <0.05, signifying statistical significance.

### 3.2 Gender‐specific prognostic factor

Acknowledging gender‐specific differences in SAH characteristics, we investigated distinct prognostic factors for each gender. Variables with *p*‐values <0.05 from the univariate analysis of male and female patients (Tables S4 and S5) were included in the multivariate analysis. For male patients, the included factors were smoking period, age, aneurysm, intracranial hematoma, IVH, midline shift, WFNS grade, HH grade, mFS score, SEBES, GCS at admission were included. For female patients, the included factors were number of children, menopause, age, hypertension, intracranial hematoma, IVH, midline shift, aneurysm, WFNS grade, HH grade, mFS grade, SEBES, GCS at admission were included. Multivariable analysis results (Tables 3 and 4) revealed that age, SEBES score, admission GCS score, and complications as independent prognostic factors for male SAH patients. In contrast, independent prognostic factors for female SAH patients encompassed age, admission GCS score, IVH, intervention, vasospasm, hydrocephalus, DCI, and epilepsy.

**TABLE 3:** Independent prognostic factors for male patients.

Variable	OR	OR (95%CI)	*p*
Age			**0.001**
<55	ref	ref	ref
≥55, <75	4.6	2.05–10.32	0.050
≥75	10.99	1–120.89	0.462
SEBES			**0.004**
0	ref	ref	ref
1	1.11	0.19–6.41	0.200
2	4.98	1.31–18.96	0.356
3	4.24	1.14–15.75	0.014
4	8.28	2.57–26.64	<0.001
Admission GCS			**<0.001**
0 (15)	ref	ref	ref
1 (13–14)	0.95	0.3–2.99	0.930
2 (9–12)	3.13	1.17–8.38	0.020
3 (3–8)	37.84	9.78–146.36	<0.001
Complications	6.91	2.99–15.96	**<0.001**

*Note:* Bolded numbers indicate *p*‐values <0.05, signifying statistical significance.

**TABLE 4:** Independent prognostic factors for female patients.

Variable	OR	OR (95%CI)	*p*
Age			**0.002**
<55	ref	ref	ref
≥55, <75	2	1.02–3.93	0.044
≥75	7.3	2.43–21.93	<0.001
Admission GCS			**<0.001**
0 (15)	ref	ref	ref
1 (13–14)	1.76	0.71–4.4	0.225
2 (9–12)	4.74	1.88–11.94	0.001
3 (3–8)	10.84	4.89–24.02	<0.001
IVH	2.01	1.07–3.76	**0.030**
Intervention			**0.008**
Others	ref	ref	ref
Clipping	1.36	0.56–3.26	0.489
Coiling	0.45	0.18–1.13	0.088
Vasospasm	2.18	1.1–4.31	**0.025**
Hydrocephalus	3.3	1.51–7.23	**0.003**
DCI	2.94	1.47–5.89	**0.002**
Epilepsy	8.91	1.5–52.93	**0.020**

*Note*: Bolded numbers indicate *p*‐values <0.05, signifying statistical significance.

### 3.3 Development and validation of gender‐specific prognosis model

Variations in independent prognostic factors among genders prompted the development of two distinct prognosis models—Model 1 for males and Model 2 for females. Internal model variables underwent evaluation for collinearity utilizing the Variance inflation factor (VIF), with criteria set to values <3 and tolerance >0.2 to exclude collinearity. Next, we depicted the prognosis models in a nomogram format, evaluating their predictive capabilities, calibration, and clinical benefit through ROC curves, calibration curve, and decision curve analysis, respectively.

**FIGURE 1:**
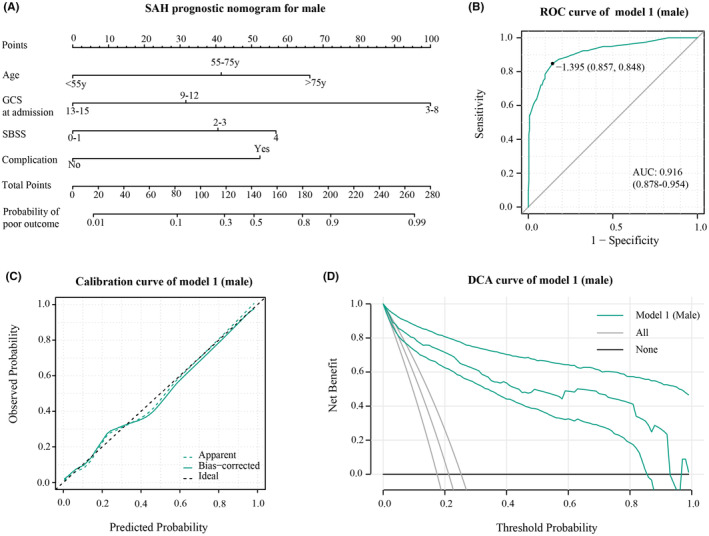
(A) Nomogram based on Model 1. (B) ROC curve of model 1 in male patients. (C) Calibration curve of model 1 in male patients. (D) DCA curve of model 1 in male patients, the reference lines represent the net benefit of the default strategy obtained when no prediction is made. None represents the scenario where all patients are considered negative, and no interventions are taken. All represents the scenario where all patients are considered positive, and interventions are always applied.

**FIGURE 2:**
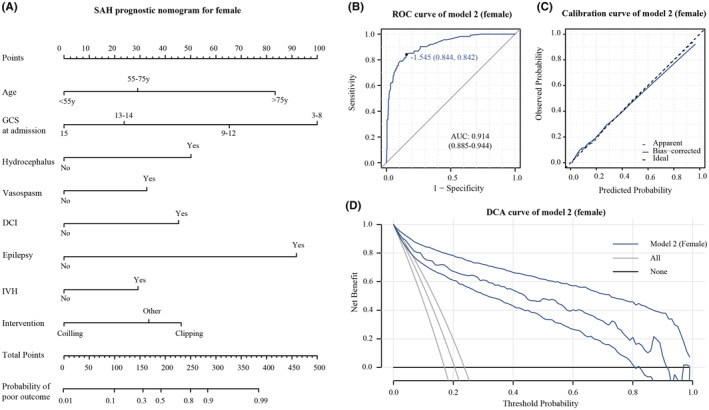
(A) Nomogram base on Model 2. (B) ROC curve of model 2 in female patients. (C) Calibration curve of model 2 in female patients. (D) DCA curve of model 2 in female patients, the reference lines represent the net benefit of the default strategy obtained when no prediction is made. None represents the scenario where all patients are considered negative, and no interventions are taken. All represents the scenario where all patients are considered positive, and interventions are always applied.

In male patients, Model 1 (Figure 1A) achieved an AUC of 0.916 (95% CI: 0.878–0.954) in the ROC curve, indicating strong discrimination (Figure 1B). The calibration curve (Figure 1C) showed a good fit between predicted and observed probabilities. Decision curve analysis (DCA) illustrated significant clinical usefulness across risk thresholds from 0.1 to 0.9 (Figure 1D). For female patients, Model 2 (Figure 2A) attained an AUC of 0.914 (95% CI: 0.885–0.944), signifying excellent predictive performance (Figure 2B). The calibration curve confirmed a good fit between predicted and observed probabilities (Figure 2C). DCA curves demonstrated substantial clinical benefits within risk thresholds from 0.1 to 0.8 (Figure 2D).

### 3.4 Further assessment of gender‐specific prognosis model

**FIGURE 3:**
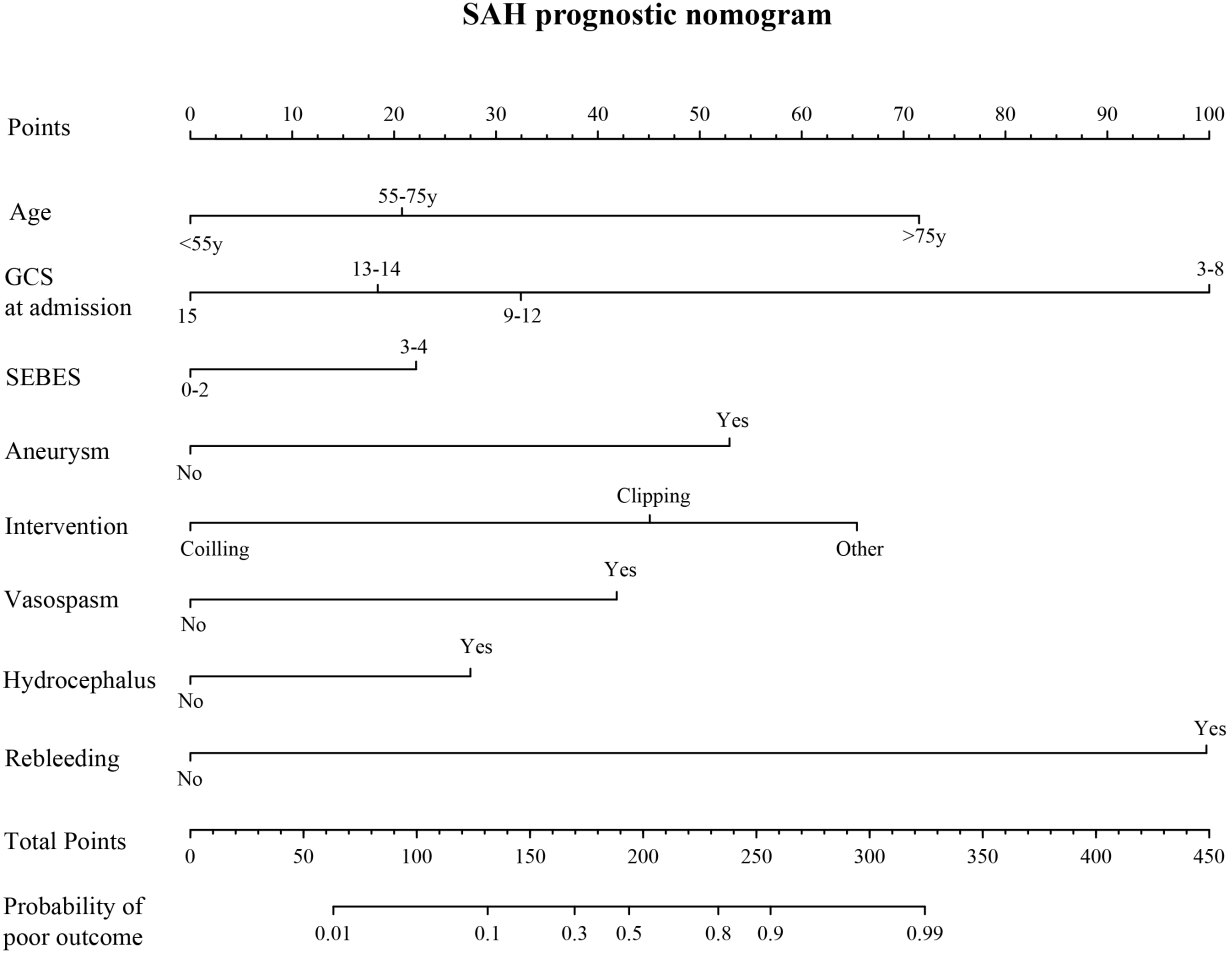
Nomogram base on Model 3.

**FIGURE 4:**
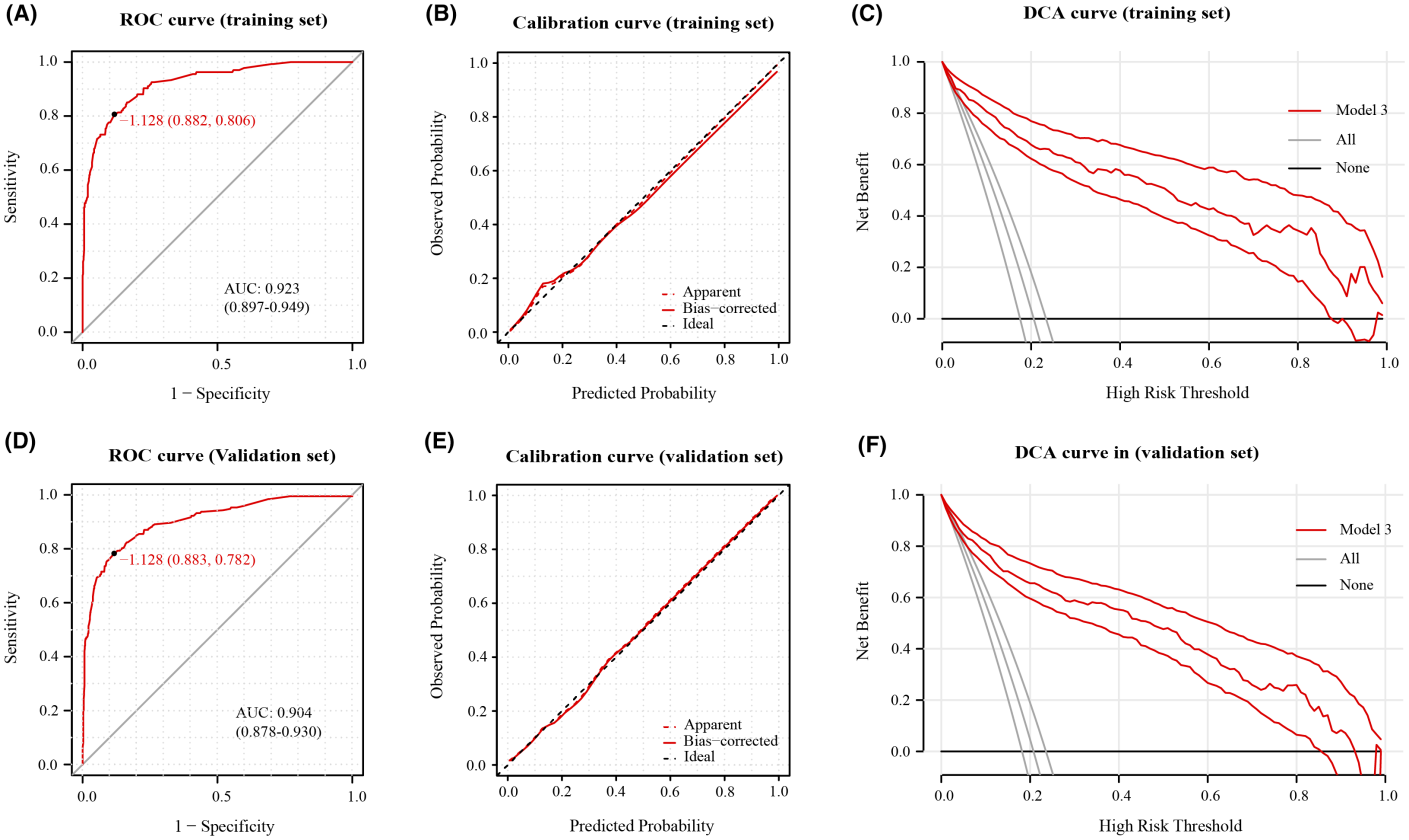
(A) ROC curve of model 3 in training set. (B) Calibration curve of model 3 in training set. (C) DCA curve of model 3 in training set. (D) ROC curve of model 3 in validation set. (E) Calibration curve of model 3 in validation set. (F) DCA curve of model 3 in validation set.

Assessment of the gender‐specific prognostic models in their respective populations displayed favorable predictive accuracy and calibration. To determine if these gender‐specific models surpass the prognosis model built on the entire cohort, we followed the same methodology to select independent prognostic factors for the entire patient cohort (Table 5) and constructed Prognosis model—Model 3 (Figure 3). With an adequate sample size in the entire cohort, we divided the population randomly in a 7:3 ratio, using 70% of patients for factor selection and model development, and the entire cohort for validation to evaluate model performance. The detailed information of patients in the 70% and 30% groups is compared and presented in Table S6. Model 3 showed good predictive accuracy and calibration in both training set and validation set (Figure 4).

**TABLE 5:** Independent prognostic factor of the entire cohort.

Variable	OR	OR (95%CI)	*p*
Age			**<0.001**
<55	ref	ref	ref
≥55, <75	2.08	1.12–3.85	0.020
≥75	12.38	3.43–44.67	<0.001
High SEBES score (3–4)	2.18	1.16–4.09	**0.020**
Admission GCS			**<0.001**
0 (15)	ref	ref	ref
1 (13–14)	1.91	0.81–4.51	0.140
2 (9–12)	3.13	1.31–7.49	0.010
3 (3–8)	33.72	14.93–76.13	<0.001
Intervention			**<0.001**
Others	ref	ref	ref
Clipping	0.49	0.11–2.12	0.339
Coiling	0.1	0.02–0.49	0.004
Vasospasm	4.36	2.38–7.99	**<0.001**
Hydrocephalus	2.63	1.29–5.38	**0.008**
Rebleeding	33.4	3.17–351.58	**0.003**
Aneurysm	6.43	1.39–29.76	**0.017**

*Note*: Bolded numbers indicate *p*‐values <0.05, signifying statistical significance.

**FIGURE 5:**
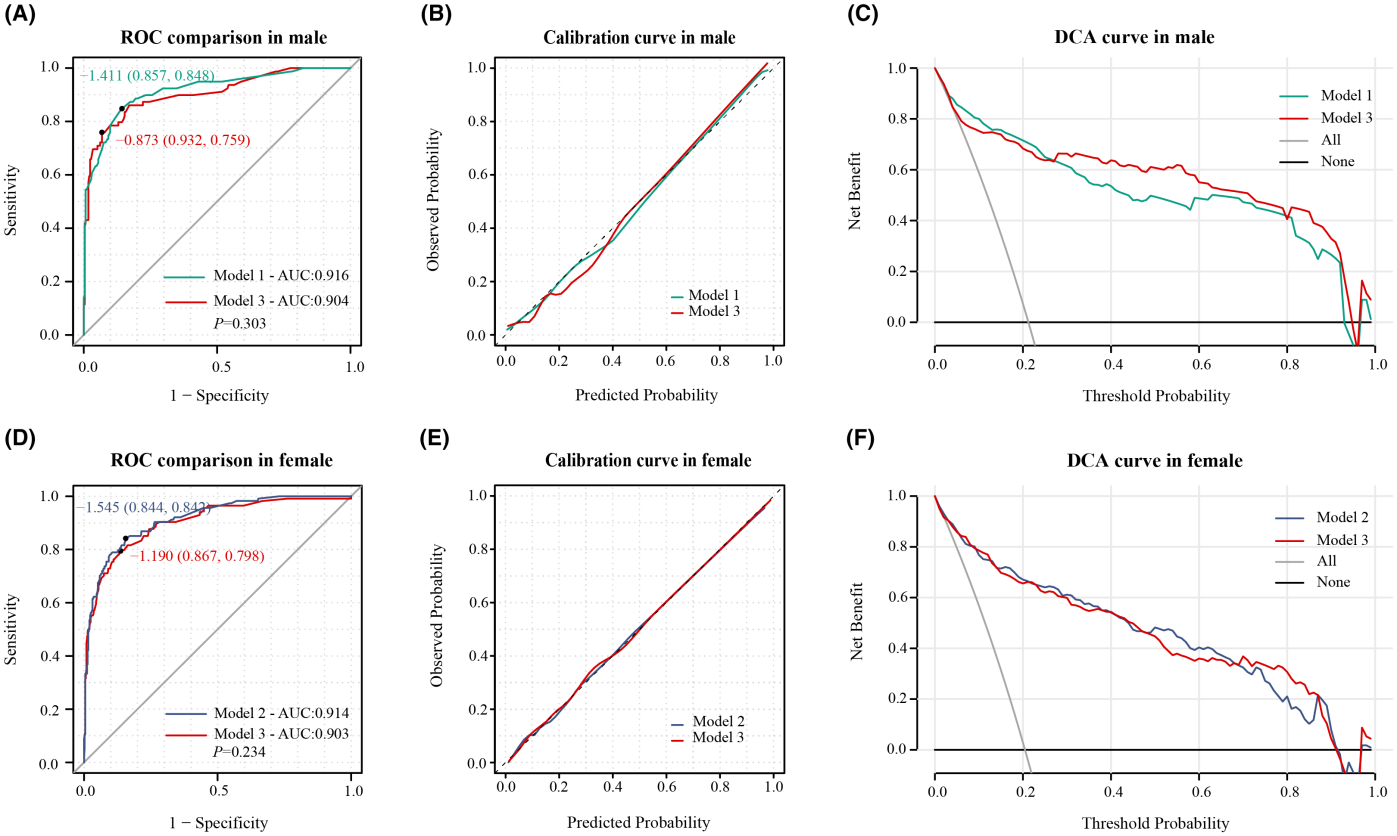
(A) ROC curve comparison of model 1 and model 3 in male patients, no significant difference in discrimination was observed (*p* = 0.303). (B) Calibration curve of model 1 and model 3 in male patients. (C) DCA curve of model 1 and model 3 in male patients. (D) ROC curve comparison of model 1 and model 3 in male patients, no significant difference in discrimination was observed (*p* = 0.303). (E) Calibration curve of model 1 and model 3 in male patients. (F) DCA curve of model 1 and model 3 in male patients.

To compare model performance, we evaluated Model 3 in male patients against Model 1 and in female patients against Model 2. In male patients, Model 1 showed similar predictive ability to Model 3 (Figure 5A), and both Model 1 and Model 3 displayed good calibration (Figure 5B). Across the risk threshold for poor prognosis from 0.1 to 0.9, both models showed clinical utility with minimal differences between them (Figure 5C). Model 2 showed similar predictive ability to Model 3 in female patients (Figure 5D). Both models exhibited good calibration in this group of patients, as indicated by calibration curve (Figure 5E). The DCA curves suggested clinical benefits for both models within poor prognosis probability thresholds of 0.1–0.8, displaying minimal differences between them (Figure 5F).

## 4 DISCUSSION

Our study uncovered variances in baseline data, disease diagnosis, and clinical treatment among male and female subarachnoid hemorrhage patients. Key findings include: (1) Gender‐based discrepancies in baseline characteristics such as age, smoking habits, hypertension history, aneurysm presence, and treatment modalities. (2) Subgroup analysis highlighted gender‐specific variations in the influence of hypertension, aneurysm characteristics, and treatment approaches on patient outcomes. (3) Development of robust gender‐specific prognostic model tailored to gender‐specific prognostic factors.

Gender disparities in neurologic diseases are increasingly evident in research. Preclinical studies indicate variations in apoptosis pathways and autophagy activity in neurons among genders under pathological conditions.^18^, ^19^, ^20^, ^21^ Moreover, gender‐specific responses of astrocytes to sex hormones contribute to stronger oxidative stress tolerance in females.^22^, ^23^, ^24^ Hormonal influences on the vascular system result in estrogen‐induced vasodilation^25^, ^26^ and testosterone‐induced vasoconstriction.^27^ Scholars advocate for viewing neurons, astrocytes, and the vascular system as an integrated neurovascular unit, crucial for maintaining stable cerebral blood flow. Recent studies by Jodie et al. and Ronney et al. have identified gender differences in cerebral blood flow regulation by this unit, influenced by patient age.^28^, ^29^ Mechanisms underlying gender disparities in the neurovascular unit involve hormones, genetics, neuro immunity, and immune responses.^30^ Recent studies have reported potential gender‐specific therapeutic approaches building on these foundations.^26^, ^31^ While gender‐specific therapeutic approaches hold promise, addressing gender disparities in baseline data, disease diagnosis, and clinical treatment, alongside identifying gender‐specific prognostic factors, is crucial for improving future therapeutic strategies.

The rupture of intracranial aneurysms is a major cause of subarachnoid hemorrhage, with females being a notable risk factor. Premenopausal women exhibit approximately 1.5 times higher aneurysm detection rates than men, while postmenopausal women show twice the rate of men.^32^ This trend is also evident in familial aneurysms.^33^ Gender differences manifest in aneurysm count, location distribution, and growth rates, attributed to disparities in cerebral vascular anatomy, hemodynamics, and hormonal influences.^34^, ^35^, ^36^ The distinct diameter and geometric shape of the Willis' circle in females subject their blood vessels to increased shear stress and peak pressure, potentially intensifying endothelial damage and fostering intracranial aneurysm formation.^35^ This contrast is particularly notable at the bifurcation of the internal carotid artery and the junction of the internal carotid artery‐posterior communicating artery.^37^ The divergence in intracranial aneurysm incidence between genders accentuates post the age of 55, concurrent with a substantial decline in estrogen levels during menopause, hinting at estrogen's potential protective role against aneurysm formation and rupture. Previous research suggests estrogen's protective mechanisms, including its influence on endothelial cell nitric oxide synthesis, modulation of cerebral artery reactivity and elasticity, mitigation of chronic arterial inflammation, and reduction of vascular oxidative stress.^38^, ^39^, ^40^, ^41^ Our analysis indicates an overall aneurysm detection rate among SAH patients of 78.15%, consistent with prior reports of 75%–80%. Furthermore, in our cohort, females (455 individuals, 81.7%) exhibited a higher ratio of detecting aneurysms than males (271 individuals, 72.8%) (*p* = 0.002). Additionally, our study uncovered a higher prevalence of anterior circulation aneurysms (within the internal carotid artery system) in both genders among SAH patients with aneurysms compared to posterior circulation (basilar artery system). However, females (180 individuals, 39.8%) showed a more pronounced presence in the posterior circulation system compared to males (53 individuals, 19.7%) (*p* < 0.001), consistent with previous findings.^42^ Nevertheless, due to our study's retrospective nature and the absence of regional sociodemographic data, comparing incidence and prevalence rates across genders is not feasible.

Our analysis highlights disparities between male and female SAH patients regarding age, smoking habits, duration of smoking, hypertension history, aneurysms, and treatment approaches. While hypertension history before disease onset is an established independent prognostic factor for SAH patients,^43^ its differential impact on prognosis across genders has not been previously documented. Notably, our analysis, for the first time, indicates that among male SAH patients, the severity of SAH, complications, and clinical prognosis appeared unrelated to a history of hypertension. Conversely, females with a hypertension history exhibited more severe SAH, increased complications, and poorer clinical prognoses. Clinically, vigilant blood pressure management and aneurysm screening are crucial for preventing SAH in hypertensive females. For female SAH patients with a hypertension history, addressing hydrocephalus timely during treatment might improve their clinical outcomes. Moreover, these observed gender‐related distinctions imply disparities in SAH progression and outcomes, despite similar baseline factors. Further investigation is warranted to determine if targeting these factors could enhance clinical intervention strategies.

In 2018, Roger et al.^36^ conducted an analysis on aneurysms in patients of different genders, focusing on their number, size, location, and morphological characteristics. They found that in females, approximately 84.62% of aneurysms were in the anterior circulation, while about 13.26% were in the posterior circulation. In males, roughly 82.39% of aneurysms were in the anterior circulation, with around 11.27% in the posterior circulation. Females showed a significantly higher likelihood of multiple aneurysms and mirror aneurysms compared to males. However, there were no significant gender‐based differences observed in aneurysm size and morphology. Similar trends were reported by Faculty et al.,^44^ although they did not find statistically significant differences. Our analysis revealed among female SAH patients a significantly higher proportion of posterior circulation aneurysms (*p* < 0.001) and a greater rate of multiple aneurysm detection (*p* = 0.022) compared to males, consistent with prior findings. Further subgroup analysis indicated a roughly similar impact of detected aneurysms on SAH patients of different genders. Aneurysmal SAH patients, in contrast to non‐aneurysmal SAH patients, displayed significantly severe conditions, a higher likelihood of craniotomy, increased clinical complications, and poorer clinical prognoses, all demonstrating statistically significant differences.

We observed a higher likelihood of male SAH patients undergoing clipping treatment, while female patients preferred coiling more often (*p* = 0.001). Irrespective of gender, patients choosing clipping often had more severe clinical conditions and increased complications. However, significant differences in prognosis between coiling and clipping were observed only in female patients. Severity of the SAH significantly influences treatment choice in clinical practice. Coiling treatment is favored for milder and stable SAH cases, while clipping is preferred for severe cases. Because clipping can not only address the aneurysm but also clears existing hematomas, reducing intracranial pressure for better prognosis. Our analysis confirmed a higher likelihood of intracranial hematomas in patients undergoing clipping, regardless of gender, supporting this scenario. Hence, the prognostic advantage of coiling over clipping in female SAH patients isn't solely due to treatment but also reflects the severity of their clinical condition. Patients needing clipping without proactive intervention may face poorer prognoses due to the more severe SAH. Conversely, in male SAH patients, despite their relative severity, those undergoing clipping didn't show significant outcome differences compared to those opting for coiling. This highlights the efficacy of clipping for SAH patients. Furthermore, these results suggest potential greater benefit from clipping in male SAH patients compared to females, a finding previously unreported. Larger sample size analyses are required to confirm this observation.

The separate prognostic factor analyses for different genders in our study revealed intriguing results. In male patients, individual complications were not independent predictors; however, collectively, various complications significantly correlated with prognosis. Conversely, in female patients, hydrocephalus, vasospasm, DCI, and rebleeding emerged as independent prognostic factors. Despite a similar overall complication incidence between genders (142 males at 38.2%; 193 females at 34.6%, *p* = 0.305), this suggests gender‐based differences in how complications impact prognosis in clinical practice. Female patients might exhibit higher susceptibility to specific complications influencing prognosis compared to males.

Patients following SAH may experience severe cerebral hypoperfusion due to rapidly rising intracranial pressure in the short term, leading to subsequent cerebral edema. Cerebral edema, a serious early stage complication post‐SAH, can be observable on CT scans but lacks a precise definition, challenging its quantitative inclusion in clinical analysis. Sung‐Ho et al. introduced the SEBES score in 2018, semi‐quantitatively defining early post‐SAH cerebral edema via CT scans, displaying good predictive capabilities for DCI occurrence.^45^ Subsequent reports associated the SEBES score with poor clinical outcome.^46^ In this study, we considered the SEBES score as a potential prognostic factor. Results indicate the SEBES score's independence as a prognostic factor in the whole cohort and male patients but not in females. Therefore, we speculate male patients' prognosis might be more affected by early cerebral edema compared to females. However, this disparity could stem from differences in SEBES score accuracy in early cerebral edema assessment across genders. A potential confounding factor is the higher age of female SAH patients in our center compared to males (*p* < 0.001). Reports suggest SEBES lacks predictive ability for DCI in elderly patients (>60 years),^47^ and no prior validations of SEBES scores based on gender groups exist. Despite this, emphasizing early cerebral edema prevention and timely treatment in male patients in clinical practice seems crucial based on our current analysis.

Previous reports highlighted an increased risk of intracerebral hemorrhage in pregnant women near delivery and postpartum.^48^ Yet, recent studies have indicated that the occurrence of subarachnoid hemorrhage does not elevate during pregnancy.^49^, ^50^ During pregnancy, significant hormonal shifts and hemodynamic fluctuations can potentially harm cerebral blood vessels,^51^ which might not exhibit immediate effects. Our analysis aimed to assess whether these factors, especially following multiple pregnancies, influence the prognosis of subarachnoid hemorrhage (SAH). In our investigation, we employed the count of children as a prognostic indicator among female patients, opting for this measure over the number of pregnancies. Initially, in univariate analysis, the number of children correlated significantly with female patient prognosis (*p* < 0.001). Even after multivariable regression, the number of children in female patients remained related to prognosis (OR: 1.65, 95% CI: 1.25–2.17, *p* < 0.001) (refer to Table S7). However, age was simultaneously excluded. Age, a well‐established independent prognostic factor for SAH, might have been excluded due to collinearity with the number of children during variable selection, as older patients typically have more children. To evaluate if the number of children independently influences prognosis regardless of age, we adjusted the female patient prognosis model for age. Consequently, the number of children ceased to be significant (OR: 1.32, 95% CI: 0.94–1.83, *p* = 0.11) (refer to Table S8). Hence, the number of children isn't genuinely an independent prognostic factor. Nonetheless, this doesn't negate the relevance of the number of pregnancies to SAH prognosis; rather, the number of children acts as a surrogate for pregnancies. Future studies necessitate more comprehensive data to validate this relationship.

Smoking independently increases the risk of intracranial aneurysm formation, growth, rupture, retreatment, and significantly raises the risk of SAH occurrence.^52^, ^53^, ^54^ A systematic review reported a heightened risk of delayed cerebral ischemia (DCI) in SAH patients who smoke (pooled OR: 1.2, 95% CI: 1.1–1.4).^55^ Smoking induces vascular damage in a time‐ and dose‐dependent manner^56^; however, the impact of this temporal and dosage correlation on SAH patient prognosis is rarely discussed.^57^, ^58^ Our study considered smoking status and self‐reported smoking duration as potential prognostic factors. Findings indicated that although smoking duration correlated significantly with male patient prognosis in univariate analysis, multivariable analysis revealed it wasn't an independent factor for SAH patient prognosis. Additionally, our study highlighted substantial differences in smoking habits and duration between female and male SAH patients. Yet, the limited number of female smokers hindered a comprehensive analysis of smoking's gender‐specific impact on SAH patients. Further exploration awaits a larger sample size in future research.

We developed and assessed two gender‐specific prognostic models based on prognostic factors, both showing favorable performance. Comparing the independent prognostic factors from female, male and entire population, revealed that most factors from entire population were a combination of gender‐specific prognostic factors. Additionally, some factors, such as the SEBSS score, showed a more detailed relationship with prognosis in the female gender‐specific model. This suggests that gender‐specific prognostic models, compared to traditional models, not only identify gender‐specific prognostic factors but also provide a clearer understanding of the relationship between prognostic factors and outcomes within each gender. Subsequently, we compared the predictive capabilities of gender‐specific prognostic models with traditional models constructed on the entire population. Model 3 was then developed within the entire population to compare predictive capabilities with gender‐specific models. Model comparisons indicated gender‐specific modeling might be preferable, despite an observed trend of enhanced predictive ability that lacked statistical significance. One explanation is the similarity in prognostic circumstances among genders. Therefore, despite some improvement in predictive ability with separate modeling, reaching a significant difference was challenging. Limited inclusion of gender‐specific factors might be another reason, restricting the models' performance enhancement through separate modeling. In the future, integrating more gender‐specific factors could notably enhance predictive ability in the models.

This study presents several limitations. Firstly, being a single‐center retrospective study, it introduces inherent biases into all statistical analyses, so it is essential to apply and extrapolate our research findings cautiously. Secondly, it lacks inclusion of several gender‐specific factors, leaving uncertainty about the potential enhancement of predictive ability through gender‐specific modeling. Thirdly, due to limited clinical data, the obstetric history of female patients couldn't be collected, leading to the use of the number of children as a substitute. While this variable showed significance in the prognosis of female SAH patients in both univariate and multivariate analyses, it lost significance after adjusting for age. Future studies should consider incorporating obstetric history to clarify the relationship between pregnancies and SAH prognosis. Fourthly, our model construction is entirely based on single‐center retrospective data, although Model 3 partially demonstrates the extrapolation ability through data splitting into training and validation groups. However, the absence of external data validation results in uncertainty regarding the interpretability of our models for data outside the center. Additionally, an analysis of aneurysm characteristics and the impact of smoking in different gender SAH patients has not been conducted. In conclusion, comprehensive research is imperative to understand the distinctions in clinical presentation, treatment strategies, prognostic factors, and clinical outcomes among SAH patients of different genders.

## AUTHOR CONTRIBUTIONS

P.X., Y.F., and J.Z. designed this study. P.X., J.W., and Y.F. collected clinical data. P.X performed statistical analysis. P.X. and X.W. wrote the original manuscript. J.Z., Z.W., K.W., and A.Z. reviewed and polished the manuscript. Y.L. handled most of the manuscript revisions. All authors approved the final manuscript.

## FUNDING INFORMATION

This study was supported by grants from the National Natural Science Foundation of China (81870916, 82071287, and 82271301) to Jianmin Zhang, National Natural Science Foundation of China (82201430) to Yuanjian Fang and Natural Science Foundation of Zhejiang Province (LGD22H090001) to Zefeng Wang.

## CONFLICT OF INTEREST STATEMENT

The authors declare that there are no conflicts of interest.

## Supporting information


Figures S1–S2.

Tables S1–S9.

## Data Availability

The data that support the findings of this study are available on request from the corresponding author. The data are not publicly available due to privacy or ethical restrictions.
