# Supplementary figures and images for: Gender‐specific prognosis models reveal differences in subarachnoid hemorrhage patients between sexes
Source: CNS Neurosci Ther. 2024 Aug 6;30(8):e14894. doi: 10.1111/cns.14894 (PMC11303446; doi:10.1111/cns.14894)

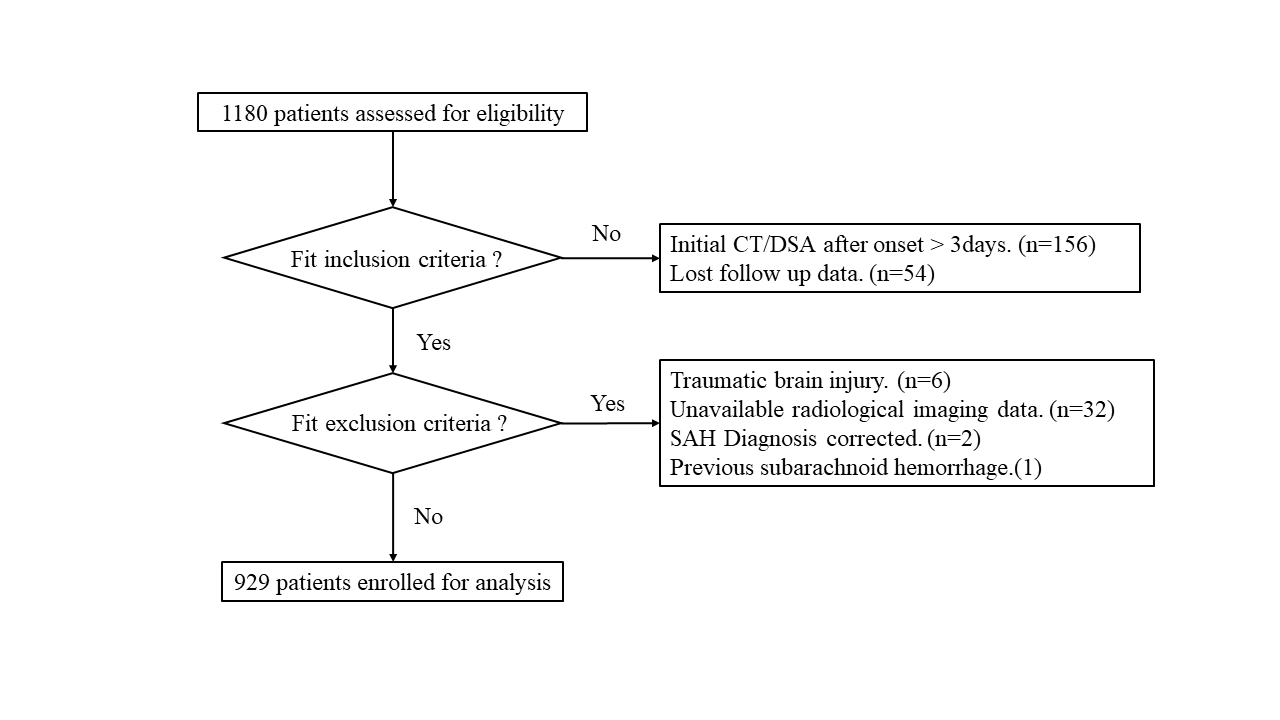

Supplement: Supplementary file 1 — Figures S1–S2. [file CNS-30-e14894-s002.zip › Fig S1.tif]

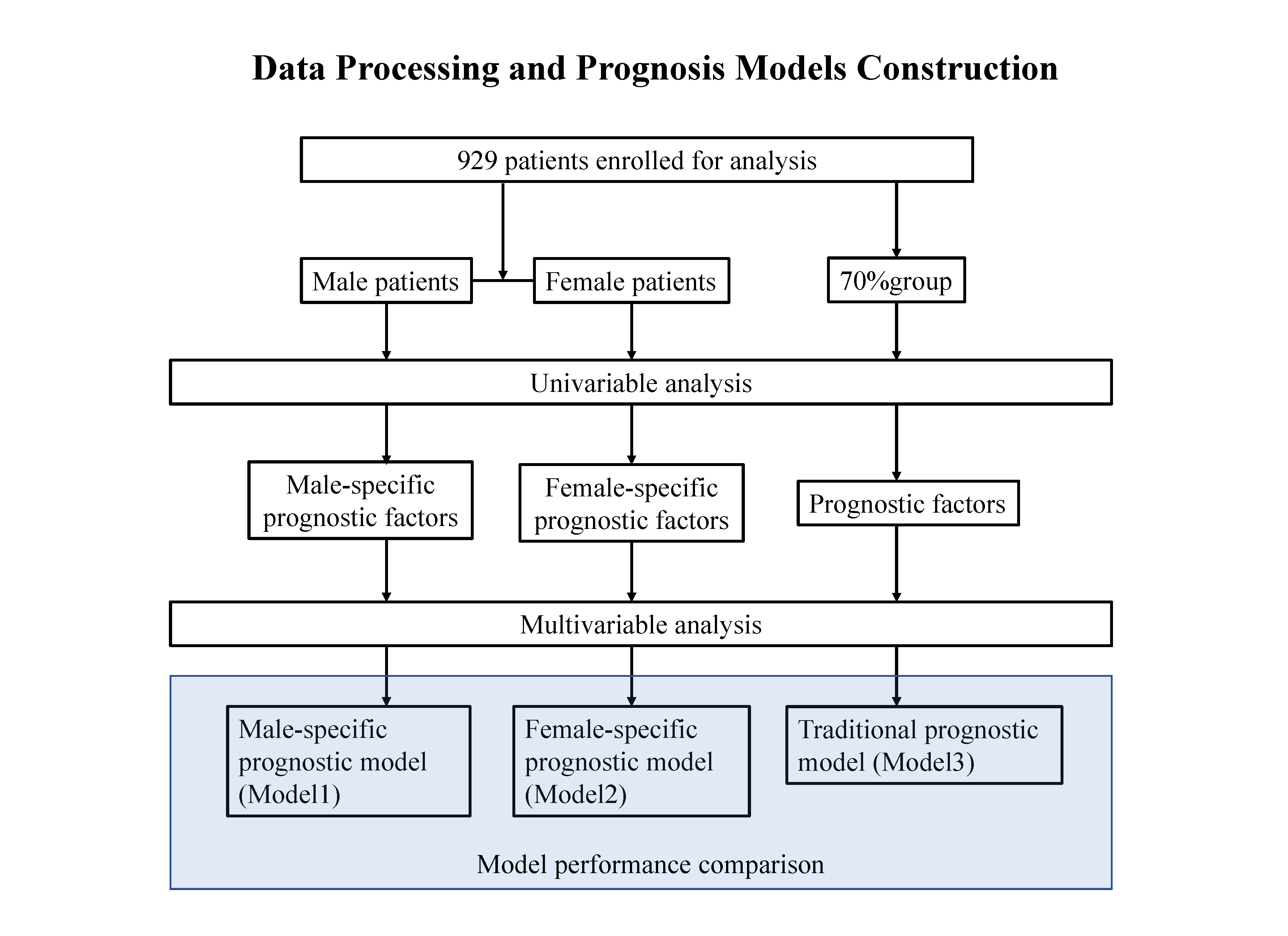

Supplement: Supplementary file 1 — Figures S1–S2. [file CNS-30-e14894-s002.zip › Fig S2 Data Processing and__ Prognosis Models Construction.tif]

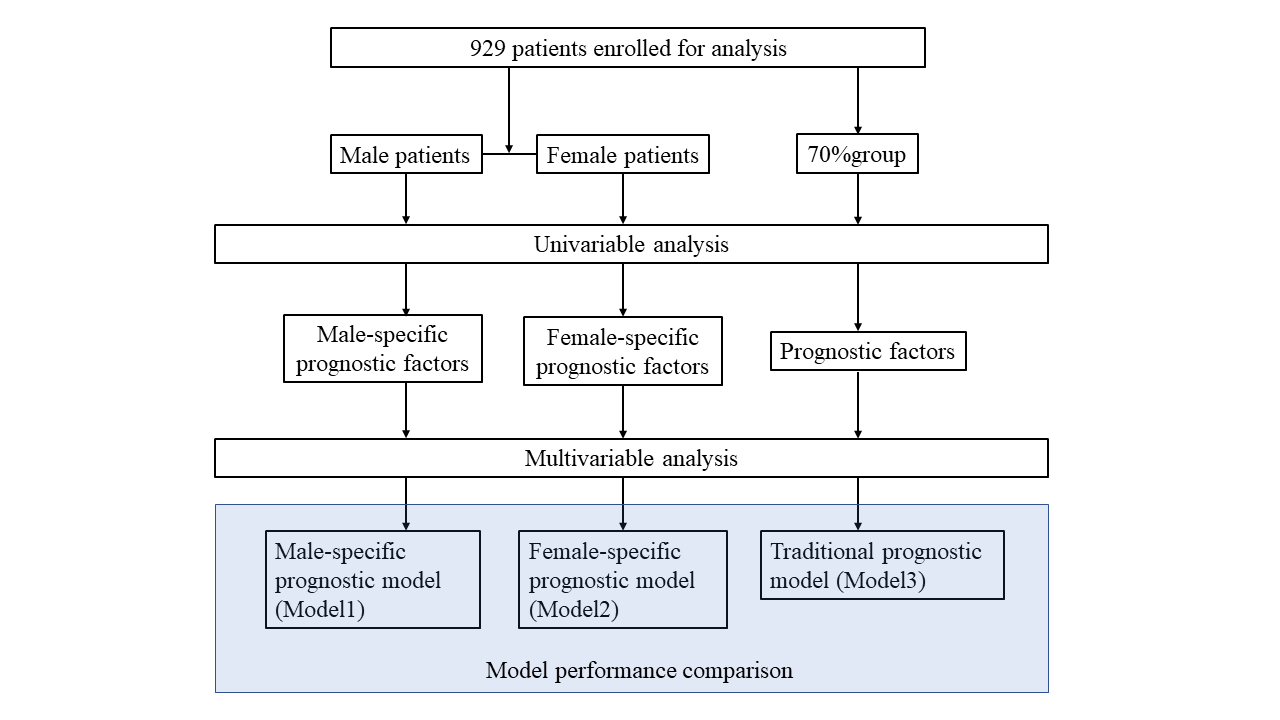

Supplement: Supplementary file 1 — Figures S1–S2. [file CNS-30-e14894-s002.zip › Fig S2.tif]
