# Supplementary material for: Gender‐specific prognosis models reveal differences in subarachnoid hemorrhage patients between sexes
Source: CNS Neurosci Ther. 2024 Aug 6;30(8):e14894. doi: 10.1111/cns.14894 (PMC11303446; doi:10.1111/cns.14894)
Supplement: Supplementary file 2 — Tables S1–S9. [file CNS-30-e14894-s001.docx]

**Supplemental materials**

| Table S1 Subgroup analysis of patients with different gender (Hypertensive VS Normotensive) | | | | | | |
| --- | --- | --- | --- | --- | --- | --- |
| Variable | Male | | *P* | Female | | *P* |
|  | Normotensive | Hypertensive |  | Normotensive | Hypertensive |  |
| Intracranial hematoma | 27 (10.1%) | 18 (17.3%) | *0.081* | 51 (15.4%) | 26 (11.6%) | *0.249* |
| IVH | 90 (33.6%) | 45 (43.3%) | *0.104* | 111 (33.4%) | 81 (36%) | *0.593* |
| Midline shift | 10 (3.7%) | 4 (3.8%) | *1.000* | 10 (3%) | 18 (8%) | ***0.014*** |
| Aneurysm | 190 (70.9%) | 81 (77.9%) | *0.219* | 265 (79.8%) | 190 (84.4%) | *0.203* |
| WFNS |  |  | *0.295* |  |  | *0.519* |
| I | 201 (75%) | 72 (69.2%) |  | 241 (72.6%) | 149 (66.2%) |  |
| II | 15 (5.6%) | 11 (10.6%) |  | 23 (6.9%) | 22 (9.8%) |  |
| III | 6 (2.2%) | 1 (1%) |  | 5 (1.5%) | 3 (1.3%) |  |
| IV | 28 (10.4%) | 15 (14.4%) |  | 34 (10.2%) | 29 (12.9%) |  |
| V | 18 (6.7%) | 5 (4.8%) |  | 29 (8.7%) | 22 (9.8%) |  |
| HH |  |  | *0.620* |  |  | *0.082* |
| 1 | 48 (17.9%) | 15 (14.4%) |  | 53 (16%) | 18 (8%) |  |
| 2 | 149 (55.6%) | 60 (57.7%) |  | 178 (53.6%) | 129 (57.3%) |  |
| 3 | 35 (13.1%) | 11 (10.6%) |  | 50 (15.1%) | 38 (16.9%) |  |
| 4 | 28 (10.4%) | 16 (15.4%) |  | 39 (11.7%) | 28 (12.4%) |  |
| 5 | 8 (3%) | 2 (1.9%) |  | 12 (3.6%) | 12 (5.3%) |  |
| mFS |  |  | *0.943* |  |  | *0.100* |
| 0 | 9 (3.4%) | 5 (4.8%) |  | 22 (6.6%) | 13 (5.8%) |  |
| 1 | 53 (19.8%) | 18 (17.3%) |  | 59 (17.8%) | 32 (14.2%) |  |
| 2 | 51 (19%) | 20 (19.2%) |  | 88 (26.5%) | 50 (22.2%) |  |
| 3 | 57 (21.3%) | 22 (21.2%) |  | 65 (19.6%) | 38 (16.9%) |  |
| 4 | 98 (36.6%) | 39 (37.5%) |  | 98 (29.5%) | 92 (40.9%) |  |
| SEBES |  |  | *0.766* |  |  | *0.492* |
| 0 | 83 (31%) | 36 (34.6%) |  | 101 (30.4%) | 61 (27.1%) |  |
| 1 | 27 (10.1%) | 12 (11.5%) |  | 36 (10.8%) | 28 (12.4%) |  |
| 2 | 44 (16.4%) | 14 (13.5%) |  | 43 (13%) | 40 (17.8%) |  |
| 3 | 36 (13.4%) | 10 (9.6%) |  | 28 (8.4%) | 20 (8.9%) |  |
| 4 | 78 (29.1%) | 32 (30.8%) |  | 124 (37.3%) | 76 (33.8%) |  |
| Admission GCS |  |  | *0.792* |  |  | *0.533* |
| 0(15) | 202 (75.4%) | 74 (71.2%) |  | 240 (72.3%) | 150 (66.7%) |  |
| 1(13-14) | 19 (7.1%) | 10 (9.6%) |  | 27 (8.1%) | 21 (9.3%) |  |
| 2(9-12) | 19 (7.1%) | 9 (8.7%) |  | 22 (6.6%) | 20 (8.9%) |  |
| 3(3-8) | 28 (10.4%) | 11 (10.6%) |  | 43 (13%) | 34 (15.1%) |  |
| Intervention |  |  | *0.520* |  |  | *0.473* |
| Clipping | 83 (31%) | 26 (25%) |  | 72 (21.7%) | 41 (18.2%) |  |
| Coiling | 105 (39.2%) | 45 (43.3%) |  | 124 (37.3%) | 94 (41.8%) |  |
| Others | 80 (29.9%) | 33 (31.7%) |  | 136 (41%) | 90 (40%) |  |
| Drainage |  |  | *0.523* |  |  | *0.537* |
| LCFD | 76 (28.4%) | 32 (30.8%) |  | 80 (24.1%) | 63 (28%) |  |
| EVD | 5 (1.9%) | 4 (3.8%) |  | 8 (2.4%) | 6 (2.7%) |  |
| Complications | 99 (36.9%) | 43 (41.3%) | *0.505* | 106 (31.9%) | 87 (38.7%) | *0.121* |
| Hydrocephalus | 34 (12.7%) | 21 (20.2%) | *0.095* | 31 (9.3%) | 35 (15.6%) | ***0.036*** |
| Vasospasm | 71 (26.5%) | 31 (29.8%) | *0.607* | 68 (20.5%) | 58 (25.8%) | *0.173* |
| DCI | 63 (23.5%) | 29 (27.9%) | *0.457* | 73 (22%) | 54 (24%) | *0.651* |
| Epilepsy | 5 (1.9%) | 2 (1.9%) | *1.000* | 3 (0.9%) | 5 (2.2%) | *0.279* |
| Rebleeding | 4 (1.5%) | 0 (0%) | *0.580* | 8 (2.4%) | 4 (1.8%) | *0.770* |
| GOS at 3 months |  |  | *0.465* |  |  | ***0.014*** |
| Favorable (4-5) | 208 (77.6%) | 85 (81.7%) |  | 276 (83.1%) | 167 (74.2%) |  |
| Poor (1-3) | 60 (22.4%) | 19 (18.3%) |  | 56 (16.9%) | 58 (25.8%) |  |

| Table S2 Subgroup analysis of patients with different gender（Without Aneurysm VS With Aneurysm） | | | | | | |
| --- | --- | --- | --- | --- | --- | --- |
| Variable | Male | | *P* | Female | | *P* |
|  | Without | With |  | Without | Aneurysm |  |
| Intracranial hematoma | 1 (1%) | 44 (16.2%) | ***<0.001*** | 5 (4.9%) | 72 (15.8%) | ***0.006*** |
| IVH | 22 (21.8%) | 113 (41.7%) | ***<0.001*** | 27 (26.5%) | 165 (36.3%) | *0.077* |
| Midline shift | 1 (1%) | 13 (4.8%) | *0.124* | 2 (2%) | 26 (5.7%) | *0.188* |
| WFNS |  |  | ***<0.001*** |  |  | ***0.008*** |
| I | 92 (91.1%) | 181 (66.8%) |  | 84 (82.4%) | 306 (67.3%) |  |
| II | 3 (3%) | 23 (8.5%) |  | 5 (4.9%) | 40 (8.8%) |  |
| III | 1 (1%) | 6 (2.2%) |  | 1 (1%) | 7 (1.5%) |  |
| IV | 1 (1%) | 42 (15.5%) |  | 3 (2.9%) | 60 (13.2%) |  |
| V | 4 (4%) | 19 (7%) |  | 9 (8.8%) | 42 (9.2%) |  |
| HH |  |  | ***<0.001*** |  |  | ***<0.001*** |
| 1 | 27 (26.7%) | 36 (13.3%) |  | 28 (27.5%) | 43 (9.5%) |  |
| 2 | 61 (60.4%) | 148 (54.6%) |  | 56 (54.9%) | 251 (55.2%) |  |
| 3 | 6 (5.9%) | 40 (14.8%) |  | 8 (7.8%) | 80 (17.6%) |  |
| 4 | 4 (4%) | 40 (14.8%) |  | 5 (4.9%) | 62 (13.6%) |  |
| 5 | 3 (3%) | 7 (2.6%) |  | 5 (4.9%) | 19 (4.2%) |  |
| mFS |  |  | ***<0.001*** |  |  | ***<0.001*** |
| 0 | 6 (5.9%) | 8 (3%) |  | 16 (15.7%) | 19 (4.2%) |  |
| 1 | 43 (42.6%) | 28 (10.3%) |  | 34 (33.3%) | 57 (12.5%) |  |
| 2 | 13 (12.9%) | 58 (21.4%) |  | 33 (32.4%) | 105 (23.1%) |  |
| 3 | 14 (13.9%) | 65 (24%) |  | 7 (6.9%) | 96 (21.1%) |  |
| 4 | 25 (24.8%) | 112 (41.3%) |  | 12 (11.8%) | 178 (39.1%) |  |
| SEBES |  |  | ***<0.001*** |  |  | ***<0.001*** |
| 0 | 59 (58.4%) | 60 (22.1%) |  | 60 (58.8%) | 102 (22.4%) |  |
| 1 | 9 (8.9%) | 30 (11.1%) |  | 10 (9.8%) | 54 (11.9%) |  |
| 2 | 10 (9.9%) | 48 (17.7%) |  | 13 (12.7%) | 70 (15.4%) |  |
| 3 | 13 (12.9%) | 33 (12.2%) |  | 2 (2%) | 46 (10.1%) |  |
| 4 | 10 (9.9%) | 100 (36.9%) |  | 17 (16.7%) | 183 (40.2%) |  |
| Admission GCS |  |  | ***<0.001*** |  |  | ***0.015*** |
| 0(15) | 92 (91.1%) | 184 (67.9%) |  | 84 (82.4%) | 306 (67.3%) |  |
| 1(13-14) | 4 (4%) | 25 (9.2%) |  | 6 (5.9%) | 42 (9.2%) |  |
| 2(9-12) | 0 (0%) | 28 (10.3%) |  | 2 (2%) | 40 (8.8%) |  |
| 3(3-8) | 5 (5%) | 34 (12.5%) |  | 10 (9.8%) | 67 (14.7%) |  |
| Intervention |  |  | ***<0.001*** |  |  | ***<0.001*** |
| Clipping | 1 (1%) | 9 (3.3%) |  | 3 (2.9%) | 215 (47.3%) |  |
| Coiling | 0 (0%) | 150 (55.4%) |  | 1 (1%) | 225 (49.5%) |  |
| Others | 100 (99%) | 112 (41.3%) |  | 98 (96.1%) | 15 (3.3%) |  |
| Drainage |  |  | ***<0.001*** |  |  | ***<0.001*** |
| LCFD | 3 (3%) | 105 (38.7%) |  | 0 (0%) | 143 (31.4%) |  |
| EVD | 1 (1%) | 8 (3%) |  | 3 (2.9%) | 11 (2.4%) |  |
| Complications | 14 (13.9%) | 128 (47.2%) | ***<0.001*** | 13 (12.7%) | 180 (39.6%) | ***<0.001*** |
| Hydrocephalus | 8 (7.9%) | 47 (17.3%) | ***0.035*** | 5 (4.9%) | 61 (13.4%) | ***0.026*** |
| Vasospasm | 10 (9.9%) | 92 (33.9%) | ***<0.001*** | 8 (7.8%) | 118 (25.9%) | ***<0.001*** |
| DCI | 10 (9.9%) | 82 (30.3%) | ***<0.001*** | 4 (3.9%) | 123 (27%) | ***<0.001*** |
| Epilepsy | 0 (0%) | 7 (2.6%) | *0.197* | 1 (1%) | 7 (1.5%) | *1.000* |
| Rebleeding | 0 (0%) | 4 (1.5%) | *0.578* | 0 (0%) | 12 (2.6%) | *0.136* |
| GOS at 3 months |  |  | ***0.002*** |  |  | ***0.023*** |
| Favorable (4-5) | 91 (90.1%) | 202 (74.5%) |  | 90 (88.2%) | 353 (77.6%) |  |
| Poor (1-3) | 10 (9.9%) | 69 (25.5%) |  | 12 (11.8%) | 102 (22.4%) |  |

| Table S3 Subgroup analysis of patients with different gender（Clipping VS Coiling） | | | | | | | |
| --- | --- | --- | --- | --- | --- | --- | --- |
| Variable | Male | | *P* | Female | | | *P* |
|  | Clipping | Coiling |  | Clipping | Coiling | |  |
| Intracranial hematoma | 31 (20.7%) | 10 (8.8%) | ***0.015*** | 50 (22.9%) | 23 (10.2%) | | ***<0.001*** |
| IVH | 62 (41.3%) | 48 (42.5%) | *0.952* | 81 (37.2%) | 81 (35.8%) | | *0.850* |
| Midline shift | 7 (4.7%) | 6 (5.3%) | *1.000* | 23 (10.6%) | 3 (1.3%) | | ***<0.001*** |
| WFNS |  |  | ***0.018*** |  |  | | *0.443* |
| I | 109 (72.7%) | 68 (60.2%) |  | 141 (64.7%) | 159 (70.4%) | |  |
| II | 13 (8.7%) | 10 (8.8%) |  | 20 (9.2%) | 19 (8.4%) | |  |
| III | 0 (0%) | 6 (5.3%) |  | 4 (1.8%) | 2 (0.9%) | |  |
| IV | 21 (14%) | 19 (16.8%) |  | 29 (13.3%) | 31 (13.7%) | |  |
| V | 7 (4.7%) | 10 (8.8%) |  | 24 (11%) | 15 (6.6%) | |  |
| HH |  |  | *0.281* |  |  | | *0.273* |
| 1 | 21 (14%) | 13 (11.5%) |  | 14 (6.4%) | 27 (11.9%) | |  |
| 2 | 89 (59.3%) | 57 (50.4%) |  | 123 (56.4%) | 124 (54.9%) | |  |
| 3 | 20 (13.3%) | 19 (16.8%) |  | 41 (18.8%) | 37 (16.4%) | |  |
| 4 | 18 (12%) | 19 (16.8%) |  | 29 (13.3%) | 31 (13.7%) | |  |
| 5 | 2 (1.3%) | 5 (4.4%) |  | 11 (5%) | 7 (3.1%) | |  |
| mFS |  |  | *0.436* |  |  | | *0.085* |
| 0 | 4 (2.7%) | 3 (2.7%) |  | 6 (2.8%) | 13 (5.8%) | |  |
| 1 | 14 (9.3%) | 11 (9.7%) |  | 24 (11%) | 30 (13.3%) | |  |
| 2 | 36 (24%) | 19 (16.8%) |  | 44 (20.2%) | 60 (26.5%) | |  |
| 3 | 39 (26%) | 25 (22.1%) |  | 54 (24.8%) | 39 (17.3%) | |  |
| 4 | 57 (38%) | 55 (48.7%) |  | 90 (41.3%) | 84 (37.2%) | |  |
| SEBES |  |  | *0.981* |  |  | | *0.586* |
| 0 | 33 (22%) | 23 (20.4%) |  | 45 (20.6%) | 55 (24.3%) | |  |
| 1 | 16 (10.7%) | 14 (12.4%) |  | 25 (11.5%) | 27 (11.9%) | |  |
| 2 | 27 (18%) | 19 (16.8%) |  | 31 (14.2%) | 39 (17.3%) | |  |
| 3 | 20 (13.3%) | 14 (12.4%) |  | 26 (11.9%) | 20 (8.8%) | |  |
| 4 | 54 (36%) | 43 (38.1%) |  | 91 (41.7%) | 85 (37.6%) | |  |
| Admission GCS |  |  | *0.055* |  |  | | *0.348* |
| 0(15) | 110 (73.3%) | 70 (61.9%) |  | 141 (64.7%) | 159 (70.4%) | |  |
| 1(13-14) | 11 (7.3%) | 14 (12.4%) |  | 22 (10.1%) | 18 (8%) | |  |
| 2(9-12) | 17 (11.3%) | 10 (8.8%) |  | 18 (8.3%) | 22 (9.7%) | |  |
| 3(3-8) | 12 (8%) | 19 (16.8%) |  | 37 (17%) | 27 (11.9%) | |  |
| Drainage |  |  | ***<0.001*** |  |  | ***<0.001*** | |
| LCFD | 97 (64.7%) | 6 (5.3%) |  | 135 (61.9%) | 8 (3.5%) | |  |
| EVD | 4 (2.7%) | 4 (3.5%) |  | 9 (4.1%) | 3 (1.3%) | |  |
| Complications | 81 (54%) | 42 (37.2%) | ***<0.001*** | 114 (52.3%) | 61 (27%) | | ***<0.001*** |
| Hydrocephalus | 25 (16.7%) | 21 (18.6%) | *0.809* | 31 (14.2%) | 25 (11.1%) | | *0.390* |
| Vasospasm | 60 (40%) | 31 (27.4%) | ***0.047*** | 73 (33.5%) | 43 (19%) | | ***0.001*** |
| DCI | 54 (36%) | 23 (20.4%) | ***<0.001*** | 86 (39.4%) | 32 (14.2%) | | ***<0.001*** |
| Epilepsy | 3 (2%) | 4 (3.5%) | *0.467* | 5 (2.3%) | 3 (1.3%) | | *0.497* |
| Rebleeding | 4 (2.7%) | 0 (0%) | *0.137* | 3 (1.4%) | 7 (3.1%) | | *0.339* |
| GOS at 3 months |  |  | *0.322* |  |  | | ***<0.001*** |
| Favorable (4-5) | 109 (72.7%) | 89 (78.8%) |  | 152 (69.7%) | 196 (86.7%) | |  |
| Poor (1-3) | 41 (27.3%) | 24 (21.2%) |  | 66 (30.3%) | 30 (13.3%) | |  |

| Table S4 Univariate analysis for male patients | | | |
| --- | --- | --- | --- |
| Variable | Favorable | Poor | *P* |
|  | N (%) | N (%) |  |
| Number of children | 2(1) | 2(1) | *0.195** |
| Smoking period (y) | 20(30) | 25(30) | ***0.030****** |
| Age |  |  | ***0.036*** |
| <55 | 163 (55.6%) | 32 (40.5%) |  |
| ≥55, <75 | 124 (42.3%) | 44 (55.7%) |  |
| ≥75 | 6 (2%) | 3 (3.8%) |  |
| Smoking situation |  |  | *0.864* |
| Current | 84 (28.7%) | 22 (27.8%) |  |
| Stopped | 12 (4.1%) | 2 (2.5%) |  |
| Never | 197 (67.2%) | 55 (69.6%) |  |
| Hypertension | 85 (29%) | 19 (24.1%) | *0.465* |
| Hyperlipidemia | 89 (30.4%) | 19 (24.1%) | *0.337* |
| Diabetes | 7 (2.4%) | 3 (3.8%) | *0.448* |
| Antiplatelet drugs | 1 (0.3%) | 0 (0%) | *1.000* |
| Heart disease | 3 (1%) | 1 (1.3%) | *1.000* |
| Aneurysm | 202 (68.9%) | 69 (87.3%) | ***0.002*** |
| Intracranial hematoma | 18 (6.1%) | 27 (34.2%) | ***<0.001*** |
| IVH | 81 (27.6%) | 54 (68.4%) | ***<0.001*** |
| Midline shift | 4 (1.4%) | 10 (12.7%) | ***0.001*** |
| WFNS |  |  | ***<0.001*** |
| I | 249 (85%) | 24 (30.4%) |  |
| II | 22 (7.5%) | 4 (5.1%) |  |
| III | 5 (1.7%) | 2 (2.5%) |  |
| IV | 15 (5.1%) | 28 (35.4%) |  |
| V | 2 (0.7%) | 21 (26.6%) |  |
| HH |  |  | ***<0.001*** |
| 1 | 57 (19.5%) | 6 (7.6%) |  |
| 2 | 191 (65.2%) | 18 (22.8%) |  |
| 3 | 32 (10.9%) | 14 (17.7%) |  |
| 4 | 12 (4.1%) | 32 (40.5%) |  |
| 5 | 1 (0.3%) | 9 (11.4%) |  |
| mFS |  |  | ***<0.001*** |
| 0 | 13 (4.4%) | 1 (1.3%) |  |
| 1 | 69 (23.5%) | 2 (2.5%) |  |
| 2 | 66 (22.5%) | 5 (6.3%) |  |
| 3 | 49 (16.7%) | 30 (38%) |  |
| 4 | 96 (32.8%) | 41 (51.9%) |  |
| SEBES |  |  | ***<0.001*** |
| 0 | 112 (38.2%) | 7 (8.9%) |  |
| 1 | 35 (11.9%) | 4 (5.1%) |  |
| 2 | 46 (15.7%) | 12 (15.2%) |  |
| 3 | 35 (11.9%) | 11 (13.9%) |  |
| 4 | 65 (22.2%) | 45 (57%) |  |
| Admission GCS |  |  | ***<0.001*** |
| 0(15) | 252 (86%) | 24 (30.4%) |  |
| 1(13-14) | 23 (7.8%) | 6 (7.6%) |  |
| 2(9-12) | 15 (5.1%) | 13 (16.5%) |  |
| 3(3-8) | 3 (1%) | 36 (45.6%) |  |
| Intervention |  |  | ***0.019*** |
| Others | 95 (32.4%) | 14 (17.7%) |  |
| Clipping | 109 (37.2%) | 41 (51.9%) |  |
| Coiling | 89 (30.4%) | 24 (30.4%) |  |
| Drainage |  |  | ***<0.001*** |
| LCFD | 85 (29%) | 23 (29.1%) |  |
| EVD | 3 (1%) | 6 (7.6%) |  |
| Complications | 74 (25.3%) | 68 (86.1%) | ***<0.001*** |
| Hydrocephalus | 24 (8.2%) | 31 (39.2%) | ***<0.001*** |
| Vasospasm | 48 (16.4%) | 54 (68.4%) | ***<0.001*** |
| DCI | 46 (15.7%) | 46 (58.2%) | ***<0.001*** |
| Epilepsy | 6 (2%) | 1 (1.3%) | *1.000* |
| Rebleeding | 0 (0%) | 4 (5.1%) | ***0.003*** |

Number of children and Smoking period are continuous variables, expressed as median (IQR). *: These variables exhibit a non-normal distribution; hence non-parametric tests are chosen for statistical analysis.

| Table S5 Univariate analysis for female patients | | | |
| --- | --- | --- | --- |
| Variable | Favorable | Poor | *P* |
|  | N (%) | N (%) |  |
| Number of children | 2(2) | 2(1) | ***<0.001****** |
| Smoking period (y) | 0(0) | 0(0) | *0.928** |
| Menopause | 297 (67.5%) | 99 (86.8%) | ***0.001*** |
| Age |  |  | ***<0.001*** |
| <55 | 201 (45.4%) | 23 (20.2%) |  |
| ≥55,<75 | 223 (50.3%) | 73 (64%) |  |
| ≥75 | 19 (4.3%) | 18 (15.8%) |  |
| Smoking situation |  |  | *0.503* |
| Current | 428 (96.6%) | 109 (95.6%) |  |
| Stopped | 3 (0.7%) | 0 (0%) |  |
| Never | 12 (2.7%) | 5 (4.4%) |  |
| Hypertension | 167 (37.7%) | 58 (50.9%) | ***0.014*** |
| Hyperlipidemia | 156 (35.2%) | 39 (34.2%) | *0.928* |
| Diabetes | 22 (5%) | 8 (7%) | *0.527* |
| Antiplatelet drugs | 2 (0.5%) | 1 (0.9%) | *0.498* |
| Heart disease | 5 (1.1%) | 2 (1.8%) | *0.636* |
| Intracranial hematoma | 31 (10%) | 24 (30.4%) | ***<0.001*** |
| IVH | 112 (25.3%) | 80 (70.2%) | ***<0.001*** |
| Midline shift | 15 (3.4%) | 13 (11.4%) | ***0.001*** |
| Aneurysm | 353 (79.7%) | 102 (89.5%) | ***0.010*** |
| WFNS |  |  | ***<0.001*** |
| I | 362 (81.7%) | 28 (24.6%) |  |
| II | 36 (8.1%) | 9 (7.9%) |  |
| III | 5 (1.1%) | 3 (2.6%) |  |
| IV | 30 (6.8%) | 33 (28.9%) |  |
| V | 10 (2.3%) | 41 (36%) |  |
| HH |  |  | ***<0.001*** |
| 1 | 67 (15.1%) | 4 (3.5%) |  |
| 2 | 285 (64.3%) | 22 (19.3%) |  |
| 3 | 60 (13.5%) | 28 (24.6%) |  |
| 4 | 28 (6.3%) | 39 (34.2%) |  |
| 5 | 3 (0.7%) | 21 (18.4%) |  |
| mFS |  |  | ***<0.001*** |
| 0 | 33 (7.4%) | 2 (1.8%) |  |
| 1 | 88 (19.9%) | 3 (2.6%) |  |
| 2 | 125 (28.2%) | 13 (11.4%) |  |
| 3 | 74 (16.7%) | 29 (25.4%) |  |
| 4 | 123 (27.8%) | 67 (58.8%) |  |
| SEBES |  |  | ***<0.001*** |
| 0 | 145 (32.7%) | 17 (14.9%) |  |
| 1 | 53 (12%) | 11 (9.6%) |  |
| 2 | 65 (14.7%) | 18 (15.8%) |  |
| 3 | 40 (9%) | 8 (7%) |  |
| 4 | 140 (31.6%) | 60 (52.6%) |  |
| Admission GCS |  |  | ***<0.001*** |
| 0(15) | 362 (81.7%) | 28 (24.6%) |  |
| 1(13-14) | 38 (8.6%) | 10 (8.8%) |  |
| 2(9-12) | 20 (4.5%) | 22 (19.3%) |  |
| 3(3-8) | 23 (5.2%) | 54 (47.4%) |  |
| Intervention |  |  | ***<0.001*** |
| Others | 95 (21.4%) | 18 (15.8%) |  |
| Clipping | 152 (34.3%) | 66 (57.9%) |  |
| Coiling | 196 (44.2%) | 30 (26.3%) |  |
| Drainage |  |  | ***<0.001*** |
| LCFD | 105 (23.7%) | 38 (33.3%) |  |
| EVD | 4 (0.9%) | 10 (8.8%) |  |
| Complications | 100 (22.6%) | 93 (81.6%) | ***<0.001*** |
| Hydrocephalus | 23 (5.2%) | 43 (37.7%) | ***<0.001*** |
| Vasospasm | 58 (13.1%) | 68 (59.6%) | ***<0.001*** |
| DCI | 57 (12.9%) | 70 (61.4%) | ***<0.001*** |
| Epilepsy | 3 (0.7%) | 5 (4.4%) | ***0.011*** |
| Rebleeding | 1 (0.2%) | 11 (9.6%) | ***<0.001*** |

Number of children and Smoking period are continuous variables, expressed as median (IQR). *: These variables exhibit a non-normal distribution; hence non-parametric tests are chosen for statistical analysis.

| Table S6 Baseline Comparison between patients in the 70% and 30% groups | | | | |
| --- | --- | --- | --- | --- |
| Variable | 70% group | | 30% group | *P* |
|  | N (%) | | N (%) |  |
| No. | | 651 | 278 |  |
| Age (y.o) | | 57(16) | 56(17) | *0.767** |
| Number of children | | 2(1) | 2(2) | *0.784** |
| Smoking period (y) | | 0(20) | 0(12.5) | *0.966** |
| Age |  | |  | *0.699* |
| <55 | 288 (44.2%) | | 131 (47.1%) |  |
| ≥55,<75 | 331 (50.8%) | | 133 (47.8%) |  |
| ≥75 | 32 (4.9%) | | 14 (5%) |  |
| Hypertension | 222 (34.1%) | | 107 (38.5%) | *0.228* |
| Hyperlipidemia | 214 (32.9%) | | 89 (32%) | *0.858* |
| Diabetes | 26 (4%) | | 14 (5%) | *0.589* |
| Antiplatelet drugs | 3 (0.5%) | | 1 (0.4%) | *1.000* |
| Heart disease | 10 (1.5%) | | 1 (0.4%) | *0.189* |
| Intracranial hematoma | 85 (13.1%) | | 37 (13.3%) | *1.000* |
| IVH | 235 (36.1%) | | 92 (33.1%) | *0.422* |
| Midline shift | 26 (4%) | | 16 (5.8%) | *0.312* |
| Aneurysm | 508 (78%) | | 218 (78.4%) | *0.966* |
| WFNS |  | |  | *0.852* |
| I | 460 (70.7%) | | 203 (73%) |  |
| II | 54 (8.3%) | | 17 (6.1%) |  |
| III | 11 (1.7%) | | 4 (1.4%) |  |
| IV | 74 (11.4%) | | 32 (11.5%) |  |
| V | 52 (8%) | | 22 (7.9%) |  |
| HH |  | |  | *0.852* |
| 1 | 460 (70.7%) | | 203 (73%) |  |
| 2 | 54 (8.3%) | | 17 (6.1%) |  |
| 3 | 11 (1.7%) | | 4 (1.4%) |  |
| 4 | 74 (11.4%) | | 32 (11.5%) |  |
| 5 | 52 (8%) | | 22 (7.9%) |  |
| mFS |  | |  | *0.110* |
| 0 | 39 (6%) | | 10 (3.6%) |  |
| 1 | 120 (18.4%) | | 42 (15.1%) |  |
| 2 | 133 (20.4%) | | 76 (27.3%) |  |
| 3 | 129 (19.8%) | | 53 (19.1%) |  |
| 4 | 230 (35.3%) | | 97 (34.9%) |  |
| SEBES |  | |  | *0.226* |
| 0 | 196 (30.1%) | | 85 (30.6%) |  |
| 1 | 67 (10.3%) | | 36 (12.9%) |  |
| 2 | 107 (16.4%) | | 34 (12.2%) |  |
| 3 | 71 (10.9%) | | 23 (8.3%) |  |
| 4 | 210 (32.3%) | | 100 (36%) |  |
| Admission GCS |  | |  | *0.308* |
| 0(15) | 463 (71.1%) | | 203 (73%) |  |
| 1(13-14) | 60 (9.2%) | | 17 (6.1%) |  |
| 2(9-12) | 45 (6.9%) | | 25 (9%) |  |
| 3(3-8) | 83 (12.7%) | | 33 (11.9%) |  |
| Intervention |  | |  | *0.197* |
| Clipping | 268 (41.2%) | | 100 (36%) |  |
| Coiling | 226 (34.7%) | | 113 (40.6%) |  |
| Others | 157 (24.1%) | | 65 (23.4%) |  |
| Drainage |  | |  | *0.165* |
| LCFD | 186 (28.4%) | | 66 (23.7%) |  |
| EVD | 20 (2.9%) | | 4 (1.4%) |  |
| None | 445 (68.4%) | | 208 (74.8%) |  |
| Complications | 232 (35.6%) | | 103 (37.1%) | *0.737* |
| Hydrocephalus | 83 (12.7%) | | 38 (13.7%) |  |
| Vasospasm | 149 (22.9%) | | 79 (28.4%) | *0.087* |
| DCI | 154 (23.7%) | | 65 (23.4%) | *0.995* |
| Epilepsy | 12 (1.8%) | | 3 (1.1%) | *0.572* |
| Rebleeding | 13 (2%) | | 3 (1.1%) | *0.417* |
| GOS at 3 months |  | |  |  |
| Favorable (4-5) | 517 (79.4%) | | 219 (78.8%) | *0.895* |
| Poor (1-3) | 134 (20.6%) | | 59 (21.2%) |  |

Age, number of children, and years of smoking are continuous variables, expressed as median (IQR). *: These variables exhibit a non-normal distribution; hence non-parametric tests are chosen for statistical analysis.

| Table S7 Multivariate logistic stepwise regression for female patients | | | |  |
| --- | --- | --- | --- | --- |
| Variable | OR | OR（95%CI） | *P* |  |
|  |  |  |  |  |
| Number of children | 1.65 | 1.25-2.17 | ***<0.001*** |  |
| Admission GCS |  |  | ***<0.001*** |  |
| 0(15) | ref | ref | *ref* |  |
| 1(13-14) | 1.73 | 0.66-4.54 | *0.311* |  |
| 2(9-12) | 6.02 | 2.39-15.14 | *<0.001* |  |
| 3(3-8) | 16.99 | 7.74-37.28 | *<0.001* |  |
| Intervention |  |  | ***0.003*** |  |
| Others | ref | ref | *ref* |  |
| Clipping | 1.46 | 0.62-3.41 | *0.370* |  |
| Coiling | 0.37 | 0.15-0.94 | *0.062* |  |
| Vasospasm | 2.36 | 1.19-4.71 | ***0.017*** |  |
| Hydrocephalus | 3.66 | 1.67-8 | ***<0.001*** |  |
| DCI | 2.8 | 1.38-5.66 | ***<0.001*** |  |
| Rebleeding | 17.27 | 1.63-182.53 | ***0.012*** |  |

| Table S8 Multivariate analysis for female patients (Age adjusted) | | | |  |
| --- | --- | --- | --- | --- |
| Variable | OR | OR（95%CI） | *P* |  |
|  |  |  |  |  |
| Number of children | 1.32 | 0.94-1.84 | *0.108* |  |
| Admission GCS |  |  | ***<0.001*** |  |
| 0(15) | ref | ref | *ref* |  |
| 1(13-14) | 1.69 | 0.65-4.39 | *0.290* |  |
| 2(9-12) | 5.92 | 2.32-15.12 | *<0.001* |  |
| 3(3-8) | 16.75 | 7.55-37.16 | *<0.001* |  |
| Intervention |  |  | ***0.005*** |  |
| Others | ref | ref | *ref* |  |
| Clipping | 1.46 | 0.62-3.45 | *0.390* |  |
| Coiling | 0.39 | 0.15-1 | *0.049* |  |
| Vasospasm | 2.43 | 1.22-4.85 | ***0.017*** |  |
| Hydrocephalus | 3.12 | 1.4-6.97 | ***0.004*** |  |
| DCI | 3.07 | 1.51-6.26 | ***0.007*** |  |
| Rebleeding | 15.62 | 1.52-160.83 | ***0.015*** |  |

| Table S9 Univariate analysis for train set | | | |
| --- | --- | --- | --- |
| Variable | Favorable | Poor | *P* |
|  | N (%) | N (%) |  |
| Gender |  |  | *0.760* |
| Male | 210 (40.6%) | 57 (42.5%) |  |
| Female | 307 (59.4%) | 77 (57.5%) |  |
| Number of children | 2(1) | 2(2) | ***<0.001****** |
| Smoking period (y) | 0(20) | 0(20) | *0.413** |
| Age |  |  | ***<0.001*** |
| <55 | 248 (48%) | 40 (29.9%) |  |
| ≥55,<75 | 251 (48.5%) | 80 (59.7%) |  |
| ≥75 | 18 (3.5%) | 14 (10.4%) |  |
| Smoking situation |  |  | *0.533* |
| Current | 360 (69.6%) | 92 (68.7%) |  |
| Stopped | 12 (2.3%) | 1 (0.7%) |  |
| Never | 145 (28%) | 41 (30.6%) |  |
| Hypertension | 352 (68.1%) | 77 (57.5%) | ***0.027*** |
| Hyperlipidemia | 347 (67.1%) | 90 (67.2%) | *1.000* |
| Diabetes | 20 (3.9%) | 6 (4.5%) | *0.942* |
| Antiplatelet drugs | 3 (0.6%) | 0 (0%) | *1.000* |
| Heart disease | 8 (1.5%) | 2 (1.5%) | *1.000* |
| Intracranial hematoma | 43 (8.3%) | 42 (31.3%) | ***<0.001*** |
| IVH | 144 (27.9%) | 91 (67.9%) | ***<0.001*** |
| Midline shift | 12 (2.3%) | 14 (10.4%) | ***<0.001*** |
| Aneurysm | 387 (74.9%) | 121 (90.3%) | ***<0.001*** |
| WFNS |  |  | ***<0.001*** |
| I | 426 (82.4%) | 34 (25.4%) |  |
| II | 44 (8.5%) | 10 (7.5%) |  |
| III | 7 (1.4%) | 4 (3%) |  |
| IV | 33 (6.4%) | 41 (30.6%) |  |
| V | 7 (1.4%) | 45 (33.6%) |  |
| HH |  |  | ***<0.001*** |
| 1 | 91 (17.6%) | 6 (4.5%) |  |
| 2 | 331 (64%) | 26 (19.4%) |  |
| 3 | 66 (12.8%) | 30 (22.4%) |  |
| 4 | 28 (5.4%) | 49 (36.6%) |  |
| 5 | 1 (0.2%) | 23 (17.2%) |  |
| mFS |  |  | ***<0.001*** |
| 0 | 36 (7%) | 3 (2.2%) |  |
| 1 | 116 (22.4%) | 4 (3%) |  |
| 2 | 125 (24.2%) | 8 (6%) |  |
| 3 | 90 (17.4%) | 39 (29.1%) |  |
| 4 | 150 (29%) | 80 (59.7%) |  |
| SEBES |  |  | ***<0.001*** |
| 0 | 181 (35%) | 15 (11.2%) |  |
| 1 | 58 (11.2%) | 9 (6.7%) |  |
| 2 | 84 (16.2%) | 23 (17.2%) |  |
| 3 | 54 (10.4%) | 17 (12.7%) |  |
| 4 | 140 (27.1%) | 70 (52.2%) |  |
| Admission GCS |  |  | ***<0.001*** |
| 0(15) | 429 (83%) | 34 (25.4%) |  |
| 1(13-14) | 46 (8.9%) | 14 (10.4%) |  |
| 2(9-12) | 24 (4.6%) | 21 (15.7%) |  |
| 3(3-8) | 18 (3.5%) | 65 (48.5%) |  |
| Intervention |  |  | ***<0.001*** |
| Others | 136 (26.3%) | 21 (15.7%) |  |
| Clipping | 187 (36.2%) | 81 (60.4%) |  |
| Coiling | 194 (37.5%) | 32 (23.9%) |  |
| Drainage |  |  | ***<0.001*** |
| LCFD | 138 (26.7%) | 478(35.1%) |  |
| EVD | 5 (1%) | 13 (10.4%) |  |
| Complications | 119 (23%) | 113 (84.3%) | ***<0.001*** |
| Hydrocephalus | 32 (6.2%) | 51 (38.1%) | ***<0.001*** |
| Vasospasm | 67 (13%) | 82 (61.2%) | ***<0.001*** |
| DCI | 76 (14.7%) | 78 (58.2%) | ***<0.001*** |
| Epilepsy | 6 (1.2%) | 6 (4.5%) | ***0.021*** |
| Rebleeding | 1 (0.2%) | 12 (9%) | ***<0.001*** |

Number of children and Smoking period are continuous variables, expressed as median (IQR). *: These variables exhibit a non-normal distribution; hence non-parametric tests are chosen for statistical analysis.
